# Anatomical Description of the Structures That Cause Tibialis Posterior Tendon Dysfunction

**DOI:** 10.7759/cureus.80196

**Published:** 2025-03-07

**Authors:** Gamze Taskin Senol, Tacettin Ayanoglu, Emre Arikan, Halil Gökkus, Abdullah Ray

**Affiliations:** 1 Anatomy, Bolu Abant İzzet Baysal University Hospital, Bolu, TUR; 2 Orthopaedics and Traumatology, Bolu Abant İzzet Baysal University Hospital, Bolu, TUR; 3 Orthopaedics and Traumatology, Bursa Yüksek İhtisas Training and Research Hospital, Bursa, TUR; 4 Orthopaedics and Traumatology, Bolu İzzet Baysal State Hospital, Bolu, TUR

**Keywords:** accessory navicular tubercle, anatomic variations, ankle, foot, magnetic resonance imaging

## Abstract

Tibialis posterior tendon (TPT) dysfunction is defined as a clinical pathology of the foot. It occurs as a sudden and progressive loss of strength due to TPT tendinopathy as a result of causes such as vascular and acute trauma or overuse. This retrospective study aimed to identify the anatomical aspects that can cause TPT dysfunction. The association between the control group and the patient group with stage 1 TPT dysfunction was assessed. The variables evaluated for this purpose were medial malleolus type, presence and type of accessory navicular tubercle, TPT width (TPTW), TPT thickness (TPTT), retromalleolar groove width (RGW), retromalleolar groove depth (RGD), retromalleolar groove angle (RGA), anterior posterior malleolar length (APML), posterior malleolar length (PML), and total posterior malleolar length (TPML). The RGA and TPML differences between the control and patient groups were determined to be statistically significant. In our opinion, identifying the risk factors for TPT dysfunction will aid in planning the therapeutic procedure.

## Introduction

In the early stages of tibialis posterior tendon (TPT) dysfunction, the process that starts with pain, sensitivity, and weakness along the tendon continues with a decrease of eccentric control in the heel strike. If dysfunction is accompanied by tenosynovitis, a large swelling occurs. In advanced stages, severe pain is observed in the tarsal sinus region. In addition, deformities begin to develop in the longitudinal arch and acquired pes planovalgus deformity develops with the weakening of the tissue and ligaments [1-4].

As mentioned above, degenerative TPT dysfunction worsens over time and progresses with new deformities. Tenosynovitis was graded by Johnson-Strom in three stages (I, II, and III) [2]. Since different deformities are associated with advanced-stage insufficiency, only patients with tenosynovitis (stage I is the earliest stage and usually begins with swelling and inflammation of the tendon (tenosynovitis)). At this stage, the structural integrity of the tendon has not yet been compromised, and early symptoms such as pain and tenderness are present. That is, there is no serious damage to the tendon at this stage, but if left untreated, it can progress to more advanced stages. This classification is important in determining the treatment approach. They were included in the study group to generate a homogeneous patient group.

The accessory navicular bone is an extra piece of bone, usually small, located in or near the TPT. Most of the time, this bone does not cause any obvious problems and is generally considered a “normal anatomical variation”. However, in some cases, especially due to trauma, overuse, or genetic factors, this bone can affect the function of the TPT, which can lead to damage to the tendon. In this study, the accessory navicular bone was considered an important factor because it is associated with TPT and may affect the function and stability of the tendon. These bone structures, also known as sesamoids, are often small, ovoid configurations, nodular. They are embedded in the tendon or joint capsule. Although sesamoid bones are usually clinically insignificant, they can also be symptomatic. If the accessory navicular bone is located within or compresses the tendon, it can compromise the structural integrity of the tendon and lead to TPT dysfunction. This can be associated with inflammation of the tendon (tenosynovitis) and other pathological changes, especially when the tendon has to bear excessive load. In addition, accessory navicular bone, especially type-III accessory navicular bone, was more frequently associated with patients with TPT dysfunction. This suggests that the presence of an accessory navicular bone may play an important role in the development of TPT dysfunction [3,4].

In studies conducted from the past to the present, the general population prevalence of accessory navicular bone has been reported as 4% to 21% in the literature [3,4]. This study aimed to investigate the anatomical factors that may cause the onset of TPT dysfunction and the classification of tuberculum navicular accessoria. For this purpose, the relationship between the patient group with stage I TPT dysfunction and the control group was evaluated. Although similar studies have been reported in the literature on different tendons [5-7], to the best of our knowledge, no studies thus far have examined the anatomical features of the surrounding region that may lead to the failure of the TPT.

## Materials and methods

Patients with ankle magnetic resonance imaging (MRI) images due to pain on the medial side of the ankle and TPT dysfunction were analyzed retrospectively using the clinical archive. As a result of clinical and radiological examinations, 61 patients with TPT dysfunction were determined as the study group. In addition, 62 patients with pain in the medial ankle but without TPT dysfunction were determined as the control group. Patients who had previous foot-ankle surgery, skin burns, skin scars, patients with cellulitis-like infections affecting the evaluation around the ankle, and those without physical examination findings and MRI archive system records were excluded from the study. The measurements were performed by a radiologist with eight years of experience in musculoskeletal radiology. They were also examined by two independent orthopedists who received six hours of training on posterior tibial tendon measurements and associated pathologies on MRI. These two independent observers were blind to the patients' clinical data. Each of them reassessed the posterior tibial tendons monthly after the first measurement and was blinded to the initial measurement results. When an experienced specialist musculoskeletal radiologist and two independent observer orthopedists were evaluated, intra- and inter-rater reliability was calculated as high correlation.

Ankle MRI studies were obtained using a 1.5 Tesla MRI device (Siemens-Magneton Symphony, Erlangen, Germany). An ankle coil was used to acquire the signals.

The duration of the MRI examination was between 15 and 20 minutes. The subjects were examined in the supine position with a 90° angle between the legs and feet. Pillows and head coils were used to stabilize the ankle in the extremity. After the "localizer," the following images were obtained for each patient on the 1.5 T-MRI system: T1-weighted spin-echo (T1W SE) images were obtained in the sagittal (repetition time (TR)/echo time (TE), 473/14; section thickness, 3 mm; field of view (FOV), 180 mm) and axial (TR/TE, 591/11; section thickness, 4 mm; FOV, 180 mm) planes. Fat-suppressed fast spin echo (FSE) T2-weighted (T2W) images were obtained in the sagittal (TR/TE, 3910/66; section thickness, 3 mm; FOV, 200 mm) and axial (TR/TE, 4100/92; section thickness, 4 mm; FOV, 190 mm) planes. Fat-suppressed proton density (PD)-weighted sequence (TR/TE, 2670/29; section thickness, 4 mm; FOV, 169 mm) was obtained in the coronal plane.

**Figure 1:**
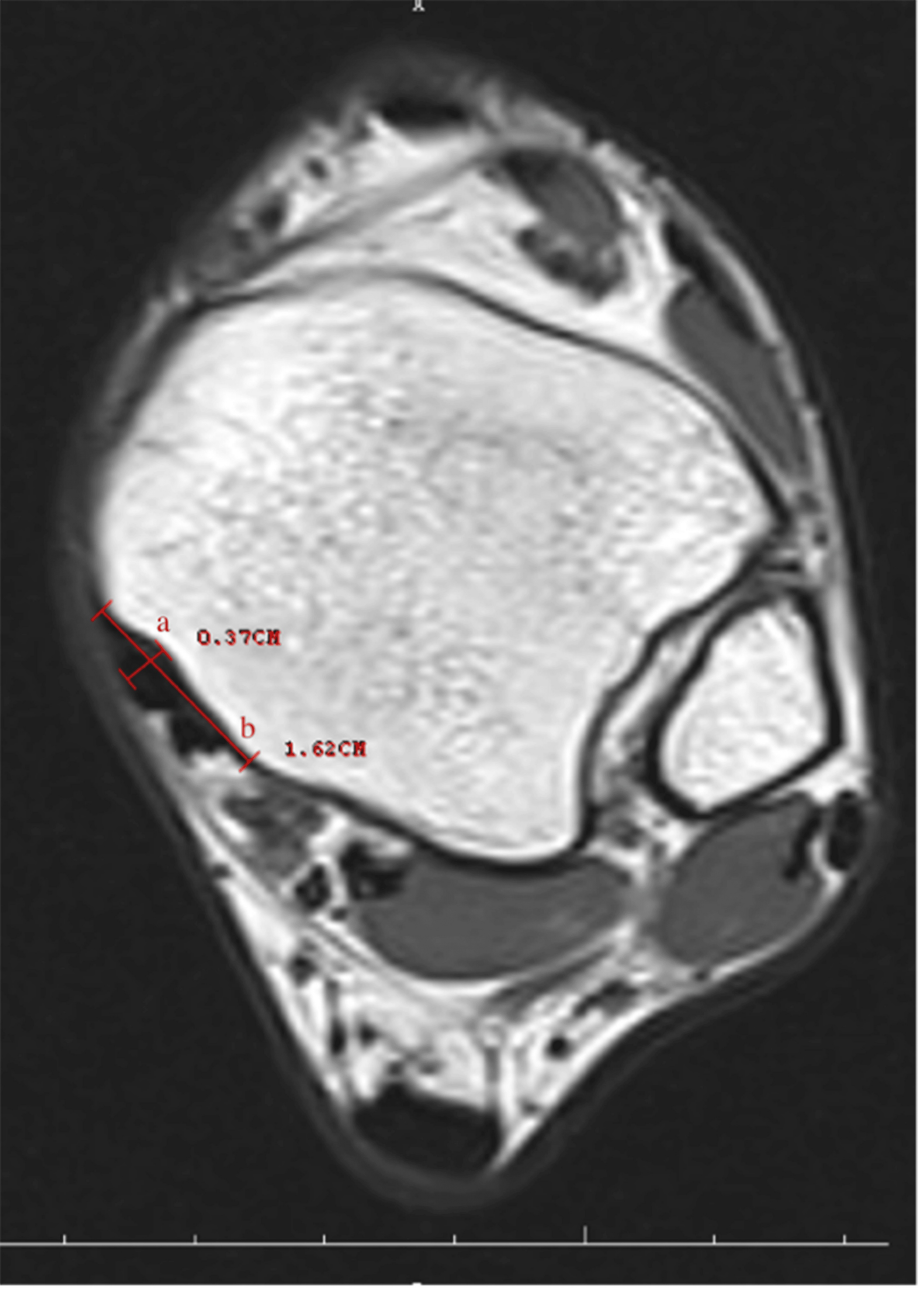
Retromalleolar groove width (a) and retromalleolar groove depth (b)

**Figure 2:**
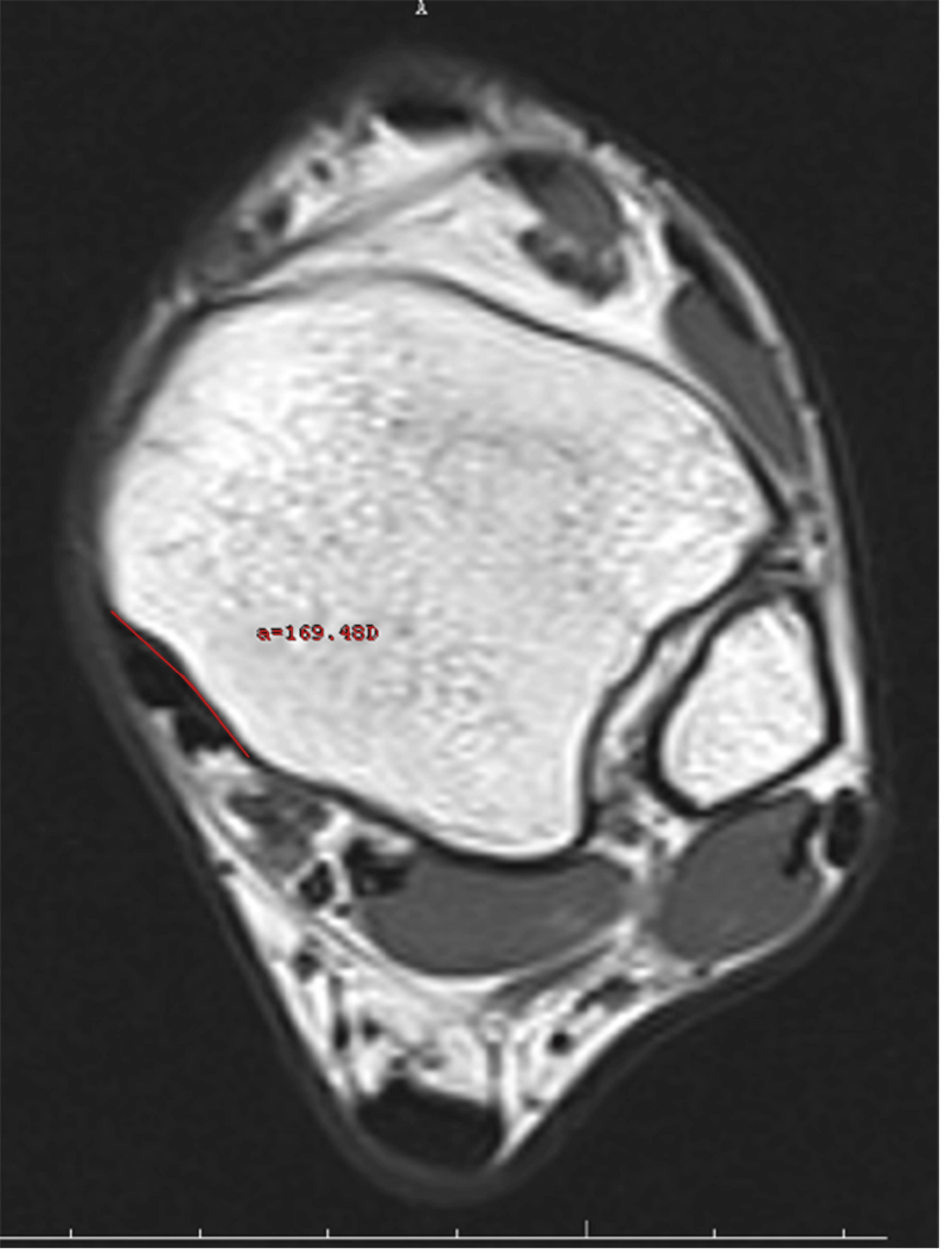
The retromalleolar groove angle

**Figure 3:**
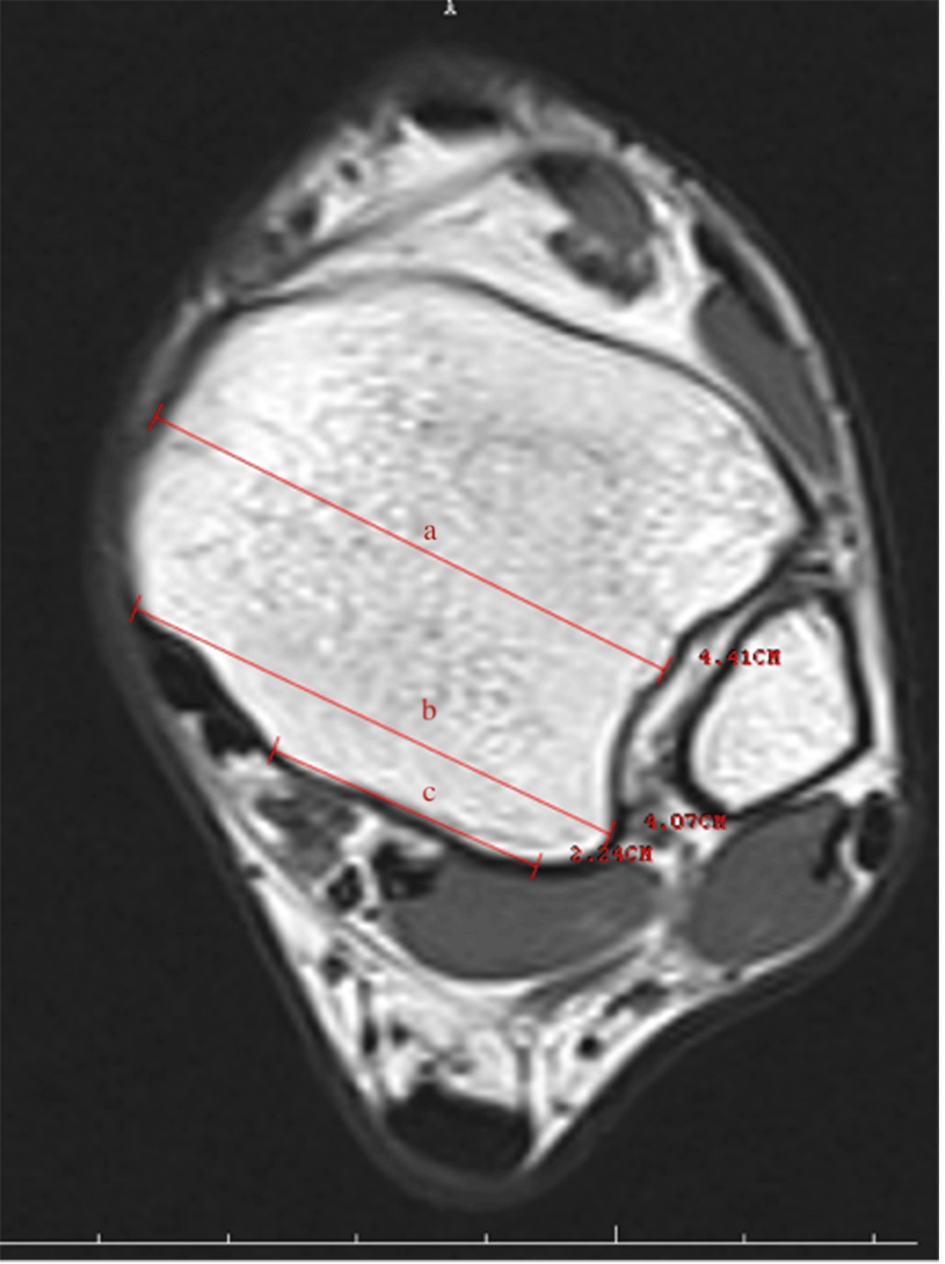
Anterior posterior malleolus length (a), total posterior malleolus length (b), and posterior malleolus length (c)

**Figure 4:**
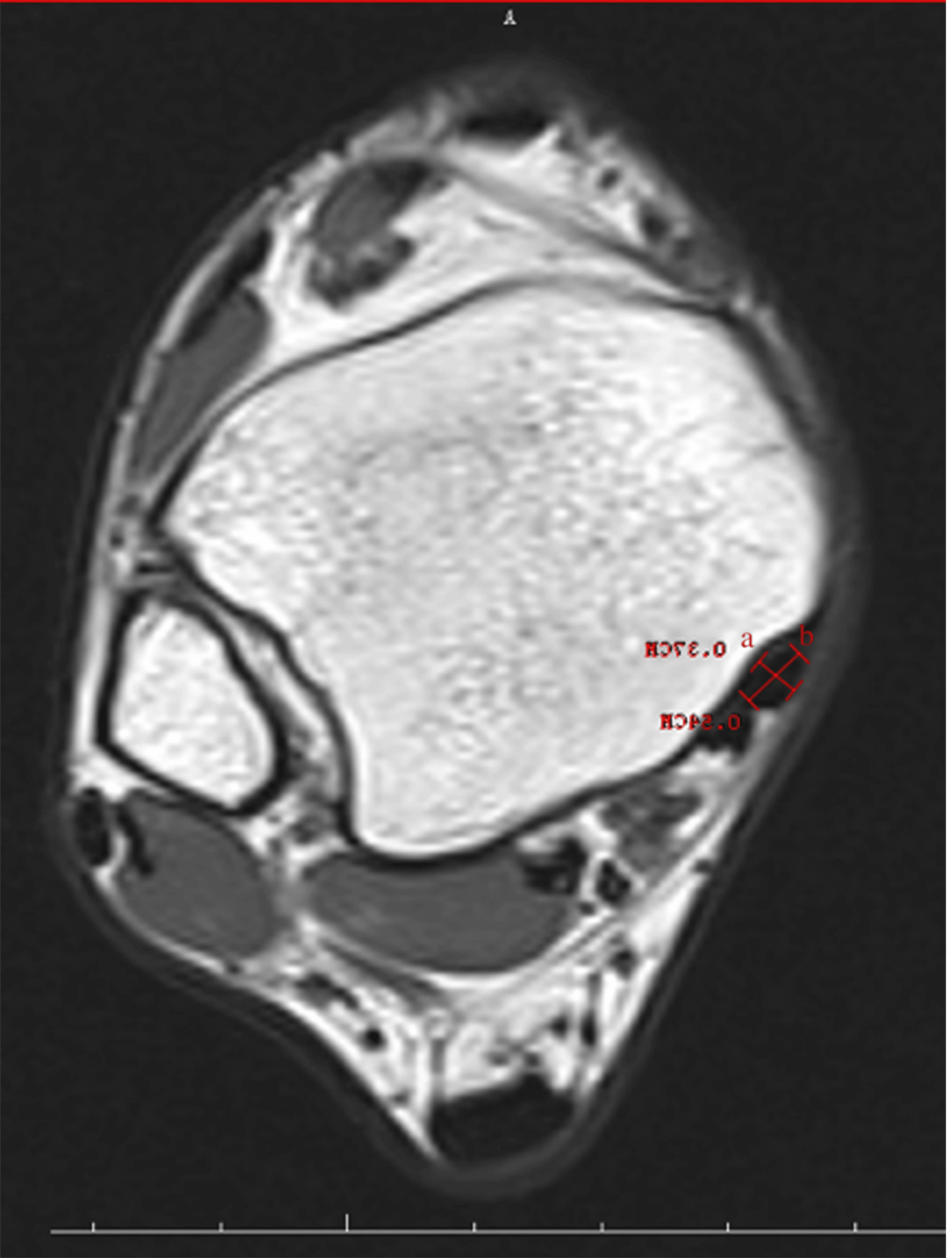
Tibialis posterior tendon thickness (a) and tibialis posterior tendon width (b)

Retromalleolar groove width (RGW) and retromalleolar groove depth (RGD) (Figure 1), retromalleolar groove angle (RGA) (Figure 2), anterior PML (APML), total PML (TPML) and posterior malleolar length (PML) (Figure 3), TPT thickness (TPTT) and TPT width (TPTW) (Figure 4), in the coronal, axial, and sagittal planes in the MRI images were evaluated.

Measurements and criteria used in the study

Measurements and criteria applied in the study were used to understand TPT dysfunction. The meaning of each measurement and criterion is as follows.

Retromalleolar Groove Width

This measurement measures the width of the groove around the medial malleolus (ankle bone). This depression is the area through which the tibialis posterior tendon passes. The width can affect the tendon's free movement and function.

Retromalleolar Groove Depth

This measurement determines the depth of the same groove. Depth can affect the placement of the tendon and anatomical changes in this area.

Retromalleolar Groove Angle

This measures the angle inside the groove. This angle can affect how the tendon moves in this area and its interaction with other structures.

Tibialis Posterior Tendon Width

This measures the width of the tibialis posterior tendon. If the tendon is wider or narrower than normal, it may affect its function and pose a risk of dysfunction.

Tibialis Posterior Tendon Thickness

This measures the thickness of the tendon. Tendon thickness is an important factor for healthy function. Thickness indicates the structural strength of the tendon. PML

Posterior Malleolar Length

This measurement determines the length at the back of the medial malleolus (ankle bone). This measurement affects the anatomical structures necessary for the tibialis posterior tendon to function correctly.

Total Posterior Malleolar Length

This measures the total length of the medial malleolus (ankle bone). This length can affect the space required for the tendon to move.

Anterior-Posterior Malleolar Length

This measures the length at the anterior part of the medial malleolus. Movement of the tendon in this area can be critical for TPT dysfunction.

Groove Depth (GD)

This measures the depth of the groove around the medial malleolus. The depth can influence how freely the tendon moves in this area and the risk of possible dysfunction.

Important criteria

These measurements used in the study are specific parameters for understanding anatomical changes that may influence the development of TPT dysfunction. For example, as the retromalleolar trough angle (RGA) widens, the tendon may be more likely to be displaced or damaged. Such measurements play an important role in identifying risk factors for TPT dysfunction and guiding the treatment process.

The medial malleolar GD and groove width (GW) measurements were performed in the axial plane at the level where the groove was the most prominent. The farthest distance between both of the edges of the groove was defined as the width, and the vertical distance drawn from this line to the deepest part of the medial malleolar groove was recorded as the depth. Both TPTW and TPTT measurements were undertaken in the same plane. The RGA was measured as the angle between the two ends of the GW and its deepest part in the axial plane. The shortest distance from the posterior border of the medial malleolar groove to the level of syndesmosis articulation in the lateral was recorded as the PML. The TPML was measured from the anterior border of the medial malleolar groove as the shortest distance from the lateral syndesmosis articulation. The measurement performed from the middle part of the tibia, where it was the thickest, was noted as the APML. There are four main shapes, comprising the omega, inverted triangle, radical sign, and wave in the medial malleolus of all normal ankles.

The statistical analyses were conducted with RStudio (2023.12.1; Posit Software, Boston, MA). A one-factor analysis of variance was applied to the variables, and the normal distribution of residuals was analyzed. The normality distribution of the variables was tested with the Anderson-Darling test. Logarithmic transformation was applied to the variables that did not conform to the normal distribution, and their conformity to the normal distribution was tested again. It was found that the transformation process was not beneficial. The mean (M) and standard deviation (SD) values were calculated for the parametric variables, while the minimum (min), maximum (max), and median values were calculated for the non-parametric variables. For the analysis of changes resulting from TPT dysfunction, independent t-tests were used for the parametric variables, while the Mann-Whitney U test was used for the non-parametric variables.

## Results

As a result of the statistical analyses, the difference between the two groups for the variables of the RGA was statistically significant. No statistically significant difference was found for the other variables. Table 1 shows the mean and SD values of the parametric variables; median, minimum, and maximum values of the non-parametric variables, as well as the p-values of the parametric variables as a result of independent t-tests and the non-parametric variables as a result of the Mann-Whitney U test.

**Table 1:** Descriptive statistics of the variables, p-values as a result of two sample t-test and Mann-Whitney U tests ^†^: p-value as a result of independent t-test; ^≠^: p-value as a result of Mann Whitney U test. ^k^: Mean±SD, ^l^: Median (Min-Max). TPTW: TPT width' TTPT: TPT thickness' GW: groove width; RGA: retromalleolar groove angle; PML: posterior malleolus length; TPML: total posterior malleolus length; APML: anterior posterior malleolus length, GD: groove depth

Variable	Type 0 (n=62)	Type 1 (n=61)	t/u-value	p-value
Age	40.7±18.8^k^	40.9±13.1	-0.065^t^	0.948^m^
TPTW (cm)	0.75 (0.50-0.92)^l^	0.74 (0.5-0.98)	2084.5^u^	0.329^n^
TTPT (cm)	0.42 (0.24-0.58)	0.41 (0.28-0.59)	1991.5^u^	0.613^n^
GW (cm)	1.49 (1.10-2.01)	1.54 (1.21-1.94)	1751^u^	0.480^n^
RA (^o^)	148 (131-176)	152 (121-171)	1600.5^u^	0.043^n^
PML (cm)	2.85 (1.14-2.74)	2.14 (1.24-2.66)	1491^u^	0.465^n^
TPML (cm)	3.54 (2.68-4.21)	3.33 (2.89-3.90	1746^u^	0.045^n^
APML (cm)	4.04 (3.21-5.12)	3.99 (3.22-5.41)	2287^u^	0.172^n^
GD (mm)	2.42±0.92	2.58±0.69	1.01^t^	0.289^m^

As per the classifications based on the results of medial malleolar types for the patient groups, 78.7% were classified as omega, 16.4% as radical, 3.3% as a wave, and 1.6% as an inverted triangle. In the control group, 59.7% were classified as omega, 22.6% as radical, 12.9% as an inverted triangle, 3.2% as a wave, and 1.6% as a triangle form. The classifications based on the result of the accessory navicular tubercle types and presence for the control and patient groups, 6.6% were type I, 3.3% were type II, 11.5% were type III in the patient group, and 3.2% were type I, and 3.2% were type III in the control group. Also, 78.7% of the patient group and 93.5% of the control group had no accessory navicular tubercles.

## Discussion

As the most important result of this study, anatomical structures such as the opening RGA and TPML were shown to be related to the formation of TPT dysfunction. Moreover, the absence of the waveform medial malleolus and the presence of type III accessory navicular tubercle were factors associated with the patient group. Among the anatomical factors evaluated in this study, it is thought that the narrowing of the TPML and the increase in the RGA cause tendon dysfunction. Typing of the medial malleolus located anterior to TPT, guiding the smooth operation of the medial malleolus fixation and deltoid ligament reconstruction, is also epidemiologically important. It describes how typological considerations on the “medial malleolus” (inner ankle bone) can affect the proper functioning of the TPT and the treatment process. This context allows for a detailed explanation of terminology and idioms. These are listed below.

Typology of the medial malleolus

The medial malleolus is the bony prominence on the inside of the ankle, at the bottom of the leg. The structural features of this prominence, its shape, and its position may differ between individuals. These differences can affect the function of the TPT and the healing processes in this area.

Keeping the TPT working properly

The tibialis posterior tendon runs along the inside of the ankle and supports the arch of the foot. Structural differences on the medial malleolus can affect the movement of the tendon in this area. If the medial malleolus does not function properly or if there is a structural defect, this can complicate the function of the tendon and lead to various disorders.

Fixing the medial malleolus and repairing the deltoid ligament

The proper functioning of the medial malleolus also affects the proper functioning of other structures in this area, such as the deltoid ligaments. The deltoid ligaments are important structures that stabilize the ankle and may need to be repaired in case of any damage. The structural features of the medial malleolus also play an important role in the repair of these ligaments.

Epidemiological significance

The “epidemiologic significance” mentioned here refers to the impact of these structural differences and treatment processes on general public health. How the structural differences of the medial malleolus have an impact on common ankle disorders, injuries, or treatment modalities in the community emphasizes the importance of research in this area. Structural differences in the medial malleolus can affect the proper functioning of the tibialis posterior tendon and deltoid ligaments. These structural features are of great importance in medical and surgical treatment processes, and a proper understanding of these differences allows treatment plans to be more effective.

In a study in which the anatomical morphological classification of the medial malleolus was conducted, 373 medial malleolus CT images were analyzed and the types determined were 66% as omega, 16% as radical, 14% as inverted triangle, and 4% as wave [8]. In the present study, while the type with the highest rate was omega in both groups, that with the lowest rate was an inverted triangle in the patient group and a triangle in the control group. 

Accessory navicular bone is a common but lesser-known anatomic variation. It may cause TPT damage in cases where it is embedded in the TPT. In general, accessory navicular bones are asymptomatic and they are present in several different anatomical sites; sometimes they may become symptomatic and present as chronic or acute foot pain [9]. When the literature was reviewed, it was seen that the incidence of accessory navicular bone is approximately between 2% and 21% in the general population [3,4,10]. While Geist reported an incidence of 14% [4], an incidence of 11.7% was reported in a study conducted on the Turkish population [11]. In the current study, it was found to be 21.2% in the patient group and 6.5% in the control group. The results are considered to be within the expected range compared to previous studies. In a study researching the incidence of accessory navicular bone in the Chinese population, 1,625 cases were examined, and navicular bone was found in 329 (20.2%). In addition, incidences were determined according to age groups, and while the highest rate was 29.7% in the 51-60 age group, the lowest rate was 0.4% in the >90 age group. In the sub-typing, 24% and 17.6% had type I, 10.9% and 10.0% had type II, and 12.5% and 9.1% had type III for women and men, respectively [12].

In the current study, in the classification of accessory navicular tubercle, 6.6% had type I, 3.3% had type II, and 11.5% had type III in the patient group, while 3.2% had type I and 3.2% had type III in the control group. Studies conducted on TPT dysfunction have usually focused on imaging and treatment methods [13-15]. There are also studies on TPT dysfunction typing in the literature. There are studies on stage I and stage II (29), those in which the stage of dysfunction was between stage I and stage III [16-19], those in which it was between stage I and stage IV [20], those in which it was only in stage II [21], and even those in which treatment was given without any staging [22]. In some studies, non-surgical approaches were recommended in asymptomatic TPT dysfunction cases, regardless of the stage. In some studies, very aggressive surgical procedures were included starting from stage I [6,23-25]. In a case-control study that was conducted, considering that leg length discrepancy may be a risk factor for TPT dysfunction, 118 patients diagnosed with TPT dysfunction and a control group of 118 individuals were evaluated. It was reported that leg length discrepancy may be a predisposing factor in terms of TPT dysfunction development [26].

This study has some limitations. Due to the study's design, a comparison of the left and right specimens was ignored. The specified anatomical insertion of the TPT may change depending on the specimens' regional and racial origins as well as the number of them that have been investigated. Another potential drawback might be that only 61 MRIs of patients were included in the study.

## Conclusions

No studies were found in the literature in which anatomical factors that may be associated with TPT dysfunction were examined. Thus, to the best of our knowledge, the present study is the first conducted in this context. Of the variables evaluated in this study, it was found that the RGA and TPML were associated in terms of stage I.

We believe that new studies should be conducted in the future with an increased number of cases. Tibialis posterior tendon dysfunction is a common but often misdiagnosed condition. It causes painful flatfoot deformity in advanced cases. Classifications in TPT dysfunction are important for choosing the best surgical treatment. We believe that the determination of predisposing factors in TPT dysfunction will aid in the planning of the treatment process.
